# Height and lung cancer risk: A meta-analysis of observational studies

**DOI:** 10.1371/journal.pone.0185316

**Published:** 2017-09-26

**Authors:** Fang Wang, Xingxiang Xu, Junjun Yang, Lingfeng Min, Sudong Liang, Yong Chen

**Affiliations:** 1 Department of Respiration, Clinical Medical School of Yangzhou University, Northern Jiangsu People’s Hospital, Yangzhou, China; 2 Department of Urology, Taizhou People's Hospital of Jiangsu Province, Taizhou, Jiangsu, China; 3 Department of Medical Oncology, Clinical Medical School of Yangzhou University, Northern Jiangsu People’s Hospital, Yangzhou, China; Dartmouth College Geisel School of Medicine, UNITED STATES

## Abstract

**Background:**

The association between height and lung cancer risk has been investigated by epidemiological studies but the results are inconsistent. This meta-analysis was to evaluate whether the height is associated with lung cancer.

**Methods:**

We identified relevant articles by searching the MEDLINE and EMBASE databases, and reviewed the reference lists of selected papers. A random effect model was used to calculate summary odds ratios (OR) and relative risk (RR) with 95% confidence intervals (95% CI). Publication bias was estimated using Egger’s regression asymmetry test.

**Results:**

We included a total 16 studies (15 prospective studies and one case–control study) on adult height and lung cancer risk in the meta-analysis. Overall, per 10-cm height increases were associated with increased risk of lung cancer (RR 1.06; 95% CI 1.03–1.09, I^2^ = 43.6%).

**Conclusions:**

In this meta-analysis, high adult height is related to increased lung cancer risk. Well-designed, large prospective studies are required to obtain a better indication of the relationship.

## Introduction

Lung cancer is one of the most common cancers worldwide in terms of both incidence and mortality [1]. Although cigarette smoking and specific occupational exposure (e.g., indoor radon, household coal smoke) are major known risk factors for lung cancer, the etiology of lung cancer remains largely elusive [2].

Adult height, which is determined by both genetic and environmental factors [3], is considered a biomarker that reflects the interplay of genetic endowment and various early-life experiences and exposures (e.g., fetal, dietary, social, and psychological circumstances) [4–8]. As the study of height can provide insights into patterns of shared and differing early determinants of major diseases of later life, it would be informative to compare the associations of adult height with subsequent risk of a wide range of diseases. Previous epidemiologic studies and meta-analyses have reported positive associations between height and risk of all cancers combined and several specific cancers, including cancer of the breast[9], prostate [10], colorectal [11], kidney [12], ovary [13], pancreas [14], testis [15], and the endometrium [16], and malignant melanoma [9, 17, 18] and lymphohematopoietic malignancies [19], and have reported negative associations between height and risk of all-cause, cardiovascular, and respiratory disease [20–25]. Numerous studies have examined the relation between height and lung cancer [9, 26]; however, the results have been inconsistent. Many factors, including selection bias and confounding, can lead to inconsistencies in such studies. However, consensus has not been reached on whether height is a risk factor for lung cancer in both women and men. To evaluate the association between height and risk of lung cancer comprehensively, we conducted a systematic review and meta-analysis of observational studies.

## Methods

### Search strategy

A systematic literature search with no language restrictions was conducted in MEDLINE and EMBASE for studies on the association between height and lung cancer incidence in humans. We searched all studies published before November 20, 2016. We used the following combinations of search terms: (“anthropometry” or “body size” or “height”) and (“lung” or “pulmonary”) and (“cancer” or “neoplasm” or “carcinoma”). In addition, we searched the reference lists of the retrieved papers for relevant articles.

### Study selection criteria

We included studies that met the following criteria: (1) case–control or cohort study investigating the association between height and lung cancer; (2) the outcome was lung cancer incidence or mortality; (3) the exposure of interest was height; and (4) reported relative risk (RR) or odds ratio (OR) estimates with their corresponding 95% confidence intervals (CIs) (or sufficient data to calculate of these effect measures). We included studies in which height had been self-reported and in which it had been directly measured. When several articles reported results from the same study population, only the most recent or comprehensive study was included.

### Data extraction

The following information was extracted from the included articles: first author’s last name, publication year, study name or source, country where the study was conducted, study period, study duration; follow-up period (cohort studies) or data collection (case–control studies), sample size(study participants, number of cases), sex, age, height assessment method (self-reported or measured), comparison of exposure level, and RRs or ORs and 95% CIs for the highest versus lowest height or per unit increase in height. If a study reported several adjustment models for potential confounding variables, we extracted the risk estimate of the most fully adjusted model. The quality of the included studies was assessed using the 9-star Newcastle Ottawa scale [27].

### Statistical analysis

We used random effect models to calculate summary RRs and 95% CIs for the highest versus lowest height and for the quantitative analyses [28]. The average natural logarithm of the RRs was estimated, and the RR from each study was weighted by the inverse of its variance. Two-tailed P ≤ 0.05 was accepted as statistically significant. In studies using height as a categorical variable, we standardized all reported RRs into comparison of the risk of the higher group with that in the lower group. Therefore, when the lowest group was not referent, we used the method proposed by Hamling et al [29] to recalculate the RRs using the lowest one as reference.

In the quantitative analyses, twelve studies [9, 23, 30–39] had directly provided RRs for per unit increase in height. For four studies [40–43] that did not provide estimate for per unit, we compute study-specific slopes (linear trends) and 95% CIs from the natural logs of the RRs and CIs across categories of height using the method by Greenland and Longnecker [44]. The method requires that the number of cases and person–years or non-cases and the relative risks with the variance estimates are known for at least three quantitative categories of use. We estimated the distribution of cases or person-years in studies that did not report these but reported the total number of cases/person-years. For example, if the total number of person-years was provided and the exposure variable was categorized by quintiles, we divided the number of person-years by five. The median or mean level of height in each category was assigned to the corresponding relative risk for each study. If a study reported height expressed as a range, we estimated the mid-point in each category by calculating the average lower and upper bound. When the highest or lowest category was open-ended, we assumed that they were of the same length as the adjacent interval.

We estimated heterogeneity among studies using the Q test and the I^2^ statistic [45]. I^2^ takes values between 0% and 100%, and I^2^> 50% is considered a measure of high heterogeneity [46]. To examine whether the results could have been influenced by a single study or a study with an extreme result, we performed sensitivity analysis by removing one study at a time. Sources of heterogeneity were explored using subgroup analyses and random-effects meta-regression analysis, according to sex, outcome (incidence and mortality), height assessment (measured and self-measured), geographic location, and adjustment for confounding factors such as smoking, alcohol, and body mass index.

We evaluated potential publication bias using funnel plots, Egger’s regression test [47], and Begg’s rank correlation test [48]. P < 0.1 was considered to indicate statistically significant publication bias. All statistical analyses were performed using Stata 12.0 (StataCorp, College Station, TX, USA).

## Results

### Literature search

**Fig 1:**
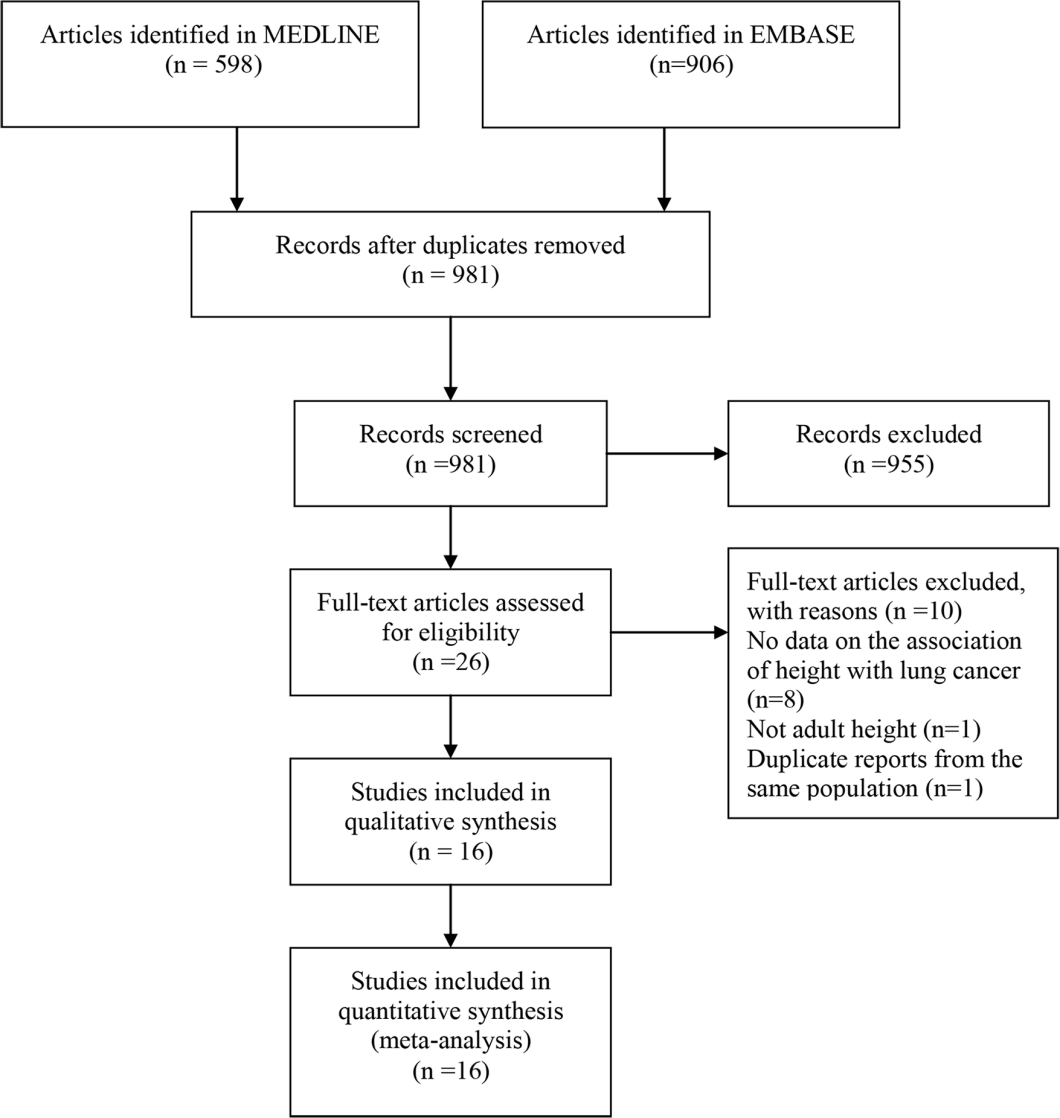
Flow diagram of the literature search.

The search retrieved 1504 publications: 598 and 906 studies from MEDLINE and EMBASE, respectively. A total 523 duplicate articles and another 955 articles were removed following the initial screening of titles and abstracts. Full-text copies of the remaining 26 potentially eligible studies were obtained. Of these, 10 were excluded because they had no data on the association of height with lung cancer risk (n = 8), did not involve adult height (n = 1), or had overlapping study populations (n = 1). Eventually, 16 studies [9, 23, 30–43] were included in the quantitative analysis, and seven [31, 32, 34, 40–43] were eligible or high versus low analysis (Fig 1).

### Study characteristics

We identified 16 studies (15 cohort studies and one case–control study) that were included in the meta-analysis of height and lung cancer risk (Table 1). The studies included a total 4,709,101 individuals, with 33,824 cases of lung cancer risk, and were published from 1981 to 2014. Five of the studies were from Europe, seven from North America, and four from Asia/Australasia. Height was measured in eleven studies and was self-reported in 5 studies. Most studies additionally adjusted for a wide range of potential risk factors: 11 for smoking [9, 30–32, 34–38, 42, 43], six for alcohol [9, 32, 34, 36, 42, 43], and seven for body mass index [9, 30–32, 37, 38, 42].

**Table 1:** Characteristics of studies included in the meta-analysis.

Author, publication year	Study name or source, location	Study period/follow-up	Study participants, sex, age	Height assessment method	Cases (no.)	Outcome	Height	RR (95% CI)	Adjustment for covariates	Study quality^a^
Albanes et al (40), 1988	National Center for Health Statistics, USA	1971-1975/ mean 10 years	12,554 (M 5,141;W7,413), aged 25–74 years	Measured	M 114	Incidence	≥177.8versus<167.6cm, M	1.10 (0.60–2.00)	Age	7
Drinkard et al (41), 1995	Iowa Women's Health Study, USA	1986-1992/mean 6 years	38,007 W, aged 55–69 years	Self-reported	233	Incidence	>165versus<155cm, W	0.81 (0.57–1.14)	Age	8
Leon et al (23), 1995	Whitehall study, UK	1967-1969/mean 18 years	18,403 M, aged 40–64 years	Measured	162	Mortality	Per 6-inch increase, M	0.89 (0.73–1.10)	Age, employment grade	7
Hebert et al (42), 1997	Physicians' Health Study (PHS), USA	1982-1995/mean 12 years	22,071 M, aged 40–84 years	Measured	170	Incidence	≥73 versus≤67 in, M	1.07 (0.63–1.83)	Age, β-carotene, BMI assignment, aspirin assignment, smoking, alcohol use, exercise frequency	8
Gunnell et al (30), 2003	Caerphilly study, UK	1979-1983/mean 21 years	2,512M, aged 45–59 years	Measured	78	Incidence	Per 6-cm increase, M	1.21 (0.96–1.51)	Father’s occupation, father’s unemployment during subject’s childhood, subject’s occupation, childhood household size, smoking history, BMI	7
Batty et al (31), 2006	Whitehall study, UK	1967–2002/maximum of 35 years	18,403 M, aged 40–64 years	Measured	801	Mortality	≥181 versus<171 cm, M Per 5-cm increase, M	1.40 (1.07–1.83) 1.08 (1.01–1.06)	Age, employment grade, physical activity, smoking habit, marital status, BMI, triceps skinfold thickness, systolic blood pressure, cholesterol, forced vital capacity, impaired glucose tolerance, diabetes, disease at entry	7
Minami et al (43), 2008	Hospital controls, Japan	1993–2007	1,730 M, aged>50 years	Self-reported	461	Incidence	≥168 versus≤159cm, M	1.04 (0.74–1.46)	Year of birth, year of survey, area of residence, referral base, smoking history, alcohol drinking history, family history of index cancer in parents and siblings, occupational history	7
Sung et al (32), 2008	Korean Adult Population Study, Korean	1994-2003/mean 8.72 years	788,789 (M 449,214;W 339,575), aged 40–64 years	Measured	4,453 M 943 W	Incidence	>171versus≤164.5cm, M >158versus≤151cm, W Per 5-cm increase, M Per 5-cm increase, W	1.18 (1.09–1.29) 1.08 (0.88–1.31) 1.07 (1.04–1.10) 1.05 (0.99–1.13)	Age, BMI, cigarette smoking, alcohol consumption, regular exercise, monthly salary level, occupation, area of residence	8
Batty et al (33), 2010	Asia Pacific Cohort Studies Collaboration (APCSC), Asia and Australasia	1961-1999/mean 5.7 years	506,648 M/W, mean age 48 years	Measured	1,226 M 332 W	Mortality	Per 6-cm increase, M Per 6-cm increase, W	1.06 (1.00–1.12) 1.08 (0.97–1.21)	Age, study, year of birth	7
Green et al (9), 2011	Million Women Study, UK	1996-2008/median 9.4 years	1,297,124 W, mean age 56.1 years	Self-reported	8,074	Incidence	Per 10-cm increase, W	1.03 (0.98–1.08)	Age, region, BMI, socioeconomic status, smoking, alcohol intake, strenuous exercise, age at menarche, parity, age at first birth	7
Ren-qiao et al (34), 2012	Shanghai women’s health study (SWHS) and Shanghai men’s health study (SMHS), China	1996-2006/mean (M 11.02; W 5.51) years	135,870 (M 61,161; W 74,709), mean age (M 54.8; W 52.1)	Measured	403 M 460 W	Incidence	≥175 versus<165 cm, M ≥162 versus<153 cm, W Per 6-cm increase, M Per 6-cm increase, W	1.55 (1.08–2.24) 1.04 (0.75–1.44) 1.11 (1.00–1.25) 1.08 (0.97–1.20)	Age, income, alcohol consumption, education, occupation, weight, ever-smoking, fruit and vegetable intake, family history of cancer, total physical activity, daily energy intake, husband’s smoking status, additional adjusted reproductive facts for women	8
Wormser et al (35), 2012	Emerging Risk Factors Collaboration (ERFC), UK	1900-1960/mean 5years	1,085,949 M/W; mean age 55 years	Measured	3,164	Mortality	Per 6.5-cm increase, M/W	1.04 (1.02–1.06)	Age, sex, year of birth, smoking	8
Walter et al (39), 2013	Vitamins and Lifestyle (VITAL) study, USA	2000-2009/mean 7.3 years	65,308 (M 32,144; W 32,894), aged 50–76 years	Self-reported	743	Incidence	per 5-inch increase, M/W	1.04 (0.90–1.19)	Age, sex, race	7
Kabat et al (36), 2013a	Women’s Health Initiative, USA	1993-2012/median 12 years	144,701 W, aged 50–79 years	Measured	1,735	Incidence	Per 10-cm increase, W	1.12 (0.92–1.38) never smoker 1.09 (1.00–1.19) ever smoker	Age, smoking, alcohol, hormone therapy, education, ethnicity, randomization status, site-specific scaling of weight/height^b^	8
Kabat et al (37), 2013b	Canadian National Breast Screening Study, Canada	1980-2000/mean 16.2 years	89,835 W, aged 40–59 years	Measured	757	Incidence	Per 10-cm increase, W	0.93 (0.82–1.06) ever smoker 1.07 (0.78–1.47) never smoker	Age at entry, menopausal status, years of education, BMI, smoking	9
Kabat et al (38), 2014c	National Institutes of Health-AARP Diet and Health Study, USA	1980-2000/mean 10.5 years	481,197(M 288,683; W 192,514), aged 50–71 years	Self-reported	6,030 M 3,486 W	Incidence	Per 10-cm increase, M Per 10-cm increase, W Per 10-cm increase, W	1.04 (1.00–1.07) 0.97 (0.92–1.02) ever smoker 1.18 (0.99–1.42) 1.14 (0.97–1.35) never smoker	Age at entry, education, race, smoking, BMI; in women, age at first menstruation	8

RR, relative risk; BMI, body mass index; W, women; M, men.

^a^Study quality was evaluated using the Newcastle–Ottawa Scale (1–9 stars).

^b^Ranged 0–3.0 in increments of 0.1.

### Analysis of high versus low height

**Fig 2:**
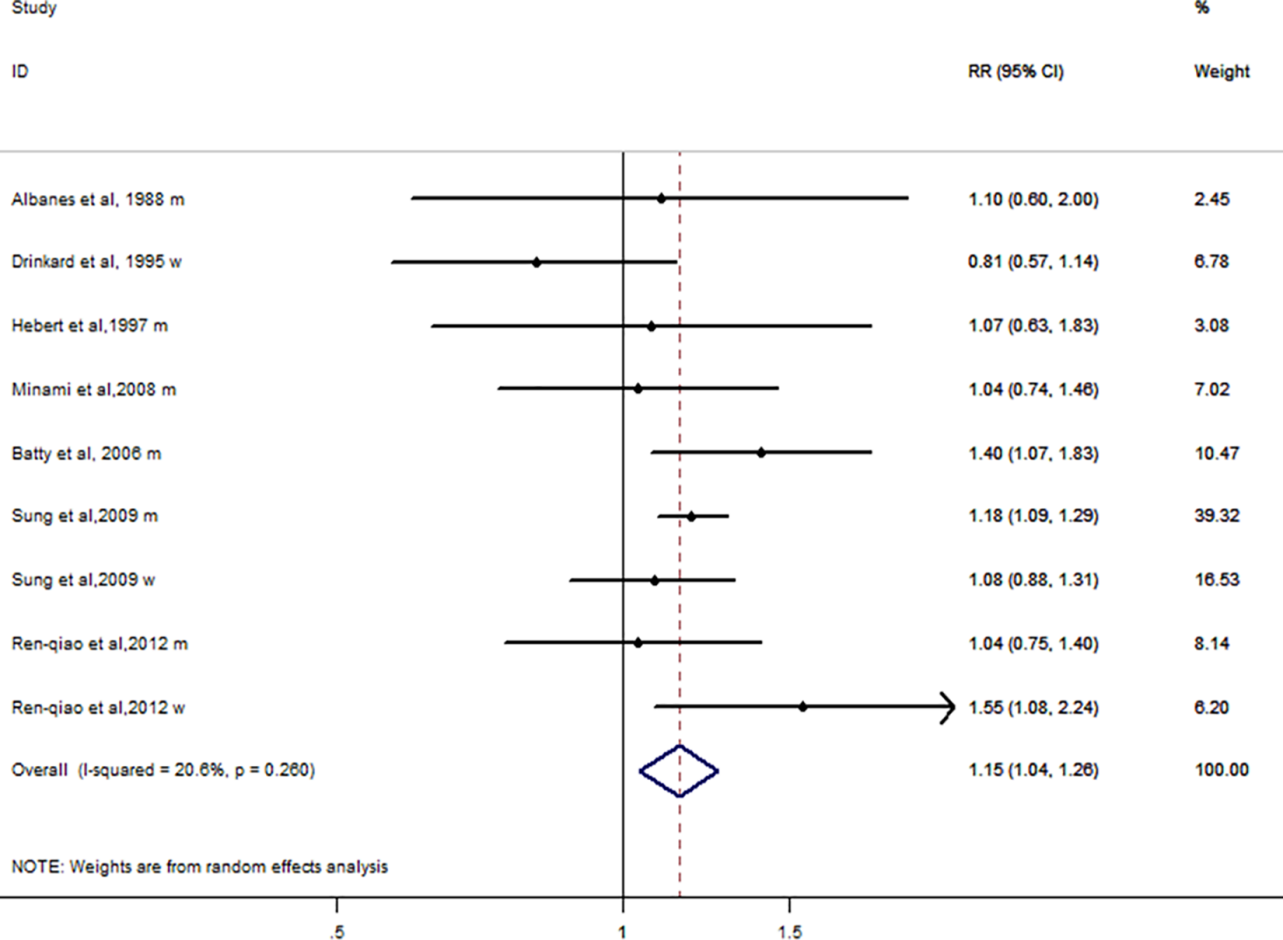
Analysis of high versus low height and lung cancer risk.

Risk of lung cancer was increased (RR = 1.15; 95% CI 1.04–1.26) in individuals with a high height compared with those with a low height (Fig 2).Heterogeneity was not statistically significant (I^2^ = 20.6%, P = 0.260) among seven studies[32–34, 40–43].

### Quantitative analysis

**Fig 3:**
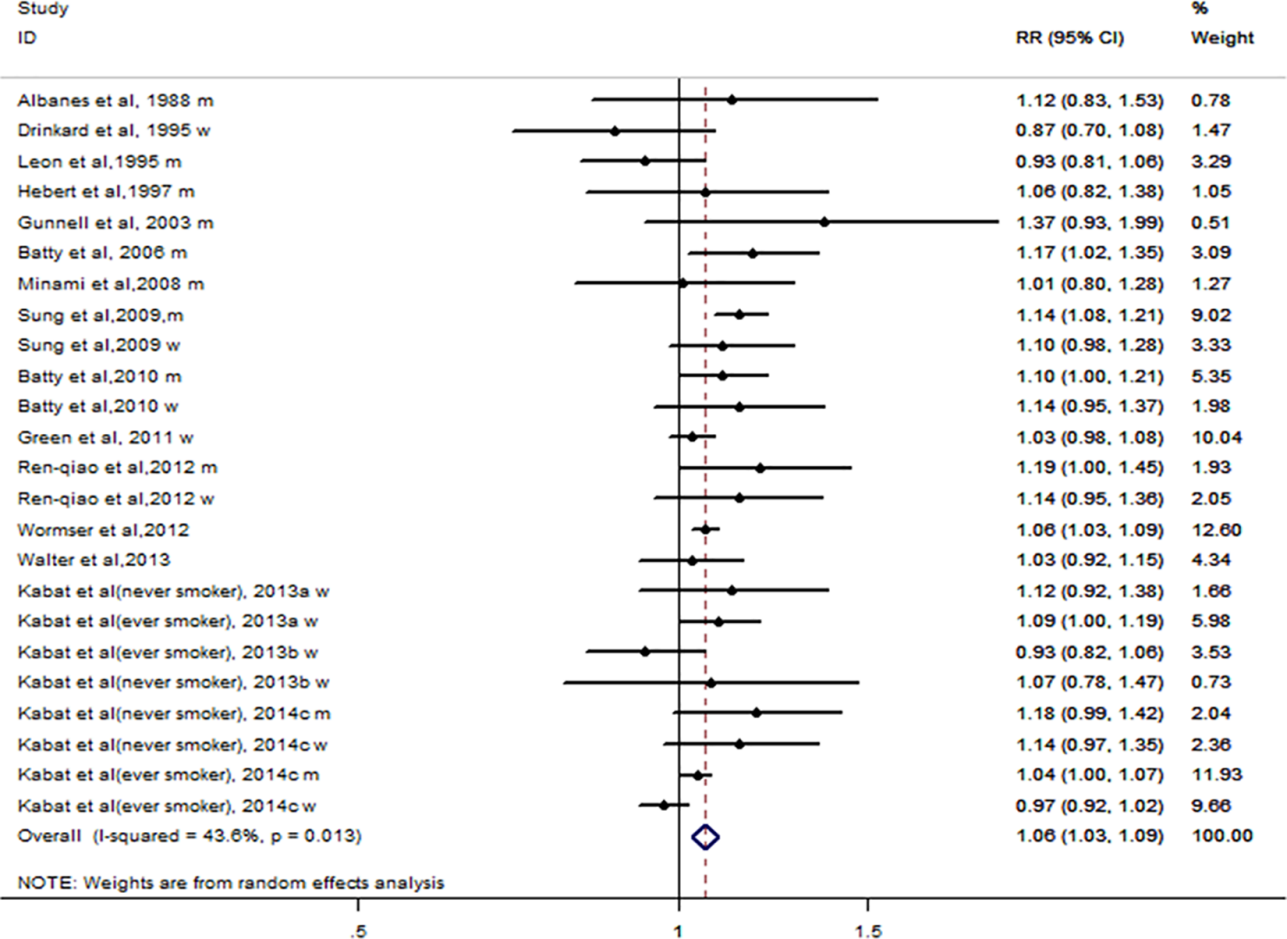
Per 10-cm height increase and lung cancer risk.

We included 16 studies [9, 23, 30–43], which included 33,824 cases among 4,709,101 participants, in the quantitative analysis. The summary RR per 10-cm height increase was 1.06 (95% CI: 1.03–1.09), with moderate heterogeneity (I^2^ = 43.6%, P = 0.013) (Fig 3).

We conducted subgroup analysis according to study design, sex, geographical area, and adjustment for confounding factors (Table 2). The results were unchanged after we had performed the meta-analysis of the cohort studies (RR 1.06; 95% CI 1.03–1.09), but not in case-control study (RR 1.01; 95% CI 0.80–1.28). A 10-cm increase in height was associated with a 9% increase in lung cancer risk in men (RR 1.09; 95% CI 1.04–1.15) and a 4% increase in women (RR 1.04; 95% CI 0.99–1.09).Excluding four studies [21, 31, 33, 35]of mortality did not affect the results(RR 1.06; 95% CI 1.02–1.09).A subgroup analysis of the assessment method of height was also performed. A significant association was observed among studies using the methods of measured (RR 1.08; 95% CI 1.05–1.12), but not among studies using the methods of self-reported (RR 1.02; 95% CI 0.99–1.06). When stratified by study location, only studies conducted in Europe (RR 1.05; 95% CI 1.00–1.10) or Asia/Australasia (RR 1.13; 95% CI 1.08–1.17) demonstrated a significant association between height and lung cancer risk, but not those conducted in North America (RR 1.03; 95% CI 0.99–1.07). The subgroup analysis results were consistent when stratified by the outcome of lung cancer and adjustment for confounding factors. Most subgroups had statistically significant heterogeneity.

**Table 2:** Quantitative subgroup analyses of height and lung cancer risk.

Subgroup	Studies (no.)	Pooled estimate	Heterogeneity	
			I^2^ (%)	P
All studies	16	1.06 (1.03–1.09)	43.6	0.013
Study design				
Case–control	1	1.01 (0.80–1.28)	-	-
Cohort	15	1.06 (1.03–1.09)	45.9	0.009
Sex				
Female	8	1.04 (0.99–1.09)	40.6	0.078
Male	10	1.09 (1.04–1.15)	44.1	0.057
Outcome				
Incidence	12	1.06 (1.02–1.09)	45.7	0.016
Mortality	4	1.07 (1.01–1.13)	40.2	0.153
Height assessment				
Measured	11	1.08 (1.05–1.12)	24.9	0.173
Self-reported	5	1.02 (0.99–1.06)	38.0	0.127
Study location				
Europe	5	1.05 (1.00–1.10)	51.7	0.082
North America	7	1.03 (0.99–1.07)	33.4	0.123
Asia/Australasia	4	1.13 (1.08–1.17)	0.0	0.943
Adjustment factors				
Smoking				
Yes	11	1.06 (1.03–1.09)	56.5	0.005
No	8	1.05 (1.00–1.12)	16.5	0.291
Alcohol				
Yes	6	1.09 (1.05–1.13)	11.8	0.336
No	10	1.04 (1.00–1.08)	48.6	0.018
Body mass index				
Yes	7	1.06 (1.01–1.11)	61.3	0.003
No	9	1.06 (1.03–1.09)	3.0	0.416

In the sensitivity analysis, the influence of each study on the pooled RR was examined by repeating the meta-analysis while omitting one study at a time. The 16 study-specific RRs ranged from1.07 (95%CI1.04–1.10) when the National Institutes of Health-AARP Diet and Health Study was excluded to1.05 (95% CI 1.02–1.08) when the Korean Adult Population Study was excluded, but were in general similar. This procedure proved that our results are reliable and robust.

**Fig 4:**
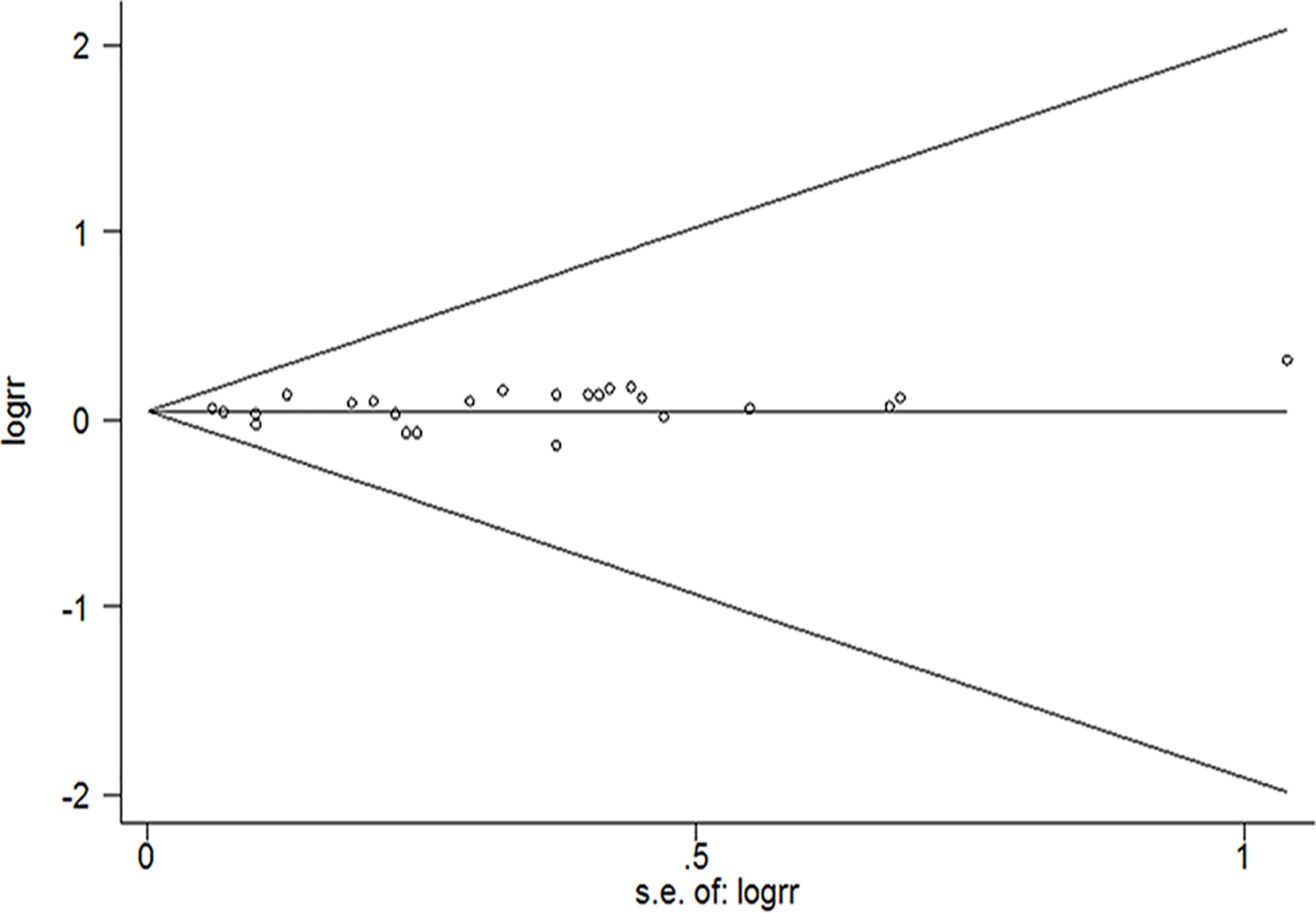
Begg’s funnel plot with pseudo-95% CI for identifying publication bias in all studies for per 10-cm height increase and lung cancer.

No indication of publication bias was observed form either with the Egger’s test (P = 0.358) or Begg’s test (P = 0.673) (Fig 4).

## Discussion

We found a similar, weakly positive association between height and lung cancer risk among men and women, although it was statistically significant only among men. Lung cancer risk increased6% for a 10-cm increase in height and a 15% increased risk for high versus low height. These associations were evident even after adjustment for smoking, alcohol, and body mass index, suggesting that height represents a robust and independent factor of increased lung cancer risk.

It has been confirmed that taller people are at higher risk for breast and colorectal cancer [9], and taller height is a possible risk factor for several other cancers; however, the potential biological mechanisms that underlie the association between greater height and lung cancer risk are unclear. It is believed that a combination of genetics, childhood and adolescent dietary factors, and infections determine adult height [8]. Previous epidemiological studies have indicated that insulin-like growth factor 1 (IGF-1) might play an important role in the development of breast [49], colorectal [50], and lung cancer [51]. IGF-1 can stimulate cell proliferation, adhesion, and migration and inhibit apoptosis, which could ultimately result in cancer. However, it is unlikely that the *IGF1* gene alone would explain the observed increased lung cancer risk associated with adult height. Other genes also revealed recently to influence adult height, such as the genes for p53, c-Myc, and estrogen receptor α (ERα), are thought to be crucial for tumorigenesis [52].

The overall analysis revealed that there was moderate heterogeneity. To investigate the potential source of heterogeneity, we carried out subgroup analysis according to study design, sex, geographical area, height assessment method, and adjustment for confounding. However, we did not find an explanation for the heterogeneity, as it persisted in most subgroup analyses. Such heterogeneity may be due other reasons not included in our subgroups, such as different adjustments for confounding factors.

Our meta-analysis has several strengths. First, it included a larger sample size (33,824 cases among 4,907,101participants) and summarized statistics which provided sufficient power to detect the association between height and lung cancer risk. Second, we used two methods to investigate the association between height and lung cancer risk, the meta-analysis by categories of height and quantitative analysis. Third, we conducted several subgroup analyses according to study characteristics, study quality scores, and adjustment for a wide range of potential confounding variables, and our findings were generally robust. Moreover, we used the Newcastle–Ottawa scale to evaluate the quality of the eligible studies, and studies included in our meta-analysis were deemed high quality because their total scores ranged 7–9.

Nevertheless, our study also has several limitations. First, although the included studies controlled for various known risk factors for lung cancer, we cannot rule out the possibility of unknown or residual confounding by dietary, behavioral, or physical activity factors. Second, the results may have been influenced by smoking, as cigarette smoke exposure has been established as an independent risk factor for developing lung cancer, but when we adjusted for smoking and age, the results did not change. Third, various height assessments were used in our analysis. Some studies used self-reporting to assess height, which may have led to overestimation of the participants’ true heights. However, the subgroup analysis revealed no substantial change in the analysis that included only the studies that relied on measured height. Finally, we cannot preclude the possibility that we might have overlooked other unpublished studies, despite our extensive literature search. The potential publication bias may have been because studies with null effects are less easily published than those reporting positive effects, therefore it was difficult for us to obtain such studies, although Egger’s test or Begg’s test did not reveal the presence of publication bias.

In conclusion, the present data suggest a positive association between height and lung cancer risk. The mechanisms involved are likely to be complex. Additional studies are warranted to extend our findings and to clarify the unknown mechanisms. In addition, given the unexplained heterogeneity, further studies are needed before a conclusion can be drawn.

## Supporting information

S1 File PRISMA 2009 flow diagram. (DOC)

S2 File PRISMA 2009 checklist. (DOC)
