# Supplementary material for: Height and lung cancer risk: A meta-analysis of observational studies
Source: PLoS One. 2017 Sep 26;12(9):e0185316. doi: 10.1371/journal.pone.0185316 (PMC5614604; doi:10.1371/journal.pone.0185316)
Supplement: S1 File — (DOC) [file pone.0185316.s001.doc]

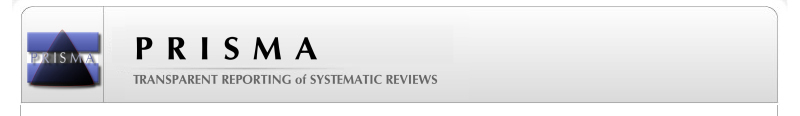
**PRISMA 2009 Flow Diagram**

**Figure 1 PRISMA flow chart for studies included in the meta-analysis.**

**Screening**

**Included**

**Eligibility**

**Identification**

Full-text articles excluded, with reasons (n =10)

No data on the association of height with lung cancer (n=8)

Not adult height (n=1)

Duplicate reports from the same population (n=1)

Articles identified in MEDLINE

(n = 598)

Articles identified in EMBASE

(n=906)

Records after duplicates removed
(n = 981)

Records screened
(n =981)

Records excluded
(n =955)

Full-text articles assessed for eligibility
(n =26)

Studies included in qualitative synthesis
(n = 16)

Studies included in quantitative synthesis (meta-analysis)
(n =16)
